# A rare cause of transient ischaemia, ST-segment elevation, and ventricular fibrillation during percutaneous lead extraction

**DOI:** 10.1093/ehjcr/ytad568

**Published:** 2023-12-08

**Authors:** Gonzalo Fernandez-Palacios, Pablo E García-Granja, Emilio García-Morán, María Sandín-Fuentes

**Affiliations:** Cardiology Department, Hospital Clínico Universitario, Valladolid, Spain; Cardiology Department, Hospital Clínico Universitario, Valladolid, Spain; CIBERCV, Cardiology Department, Hospital Clínico Universitario, Valladolid, Spain; Cardiology Department, Hospital Clínico Universitario, Valladolid, Spain; Cardiology Department, Hospital Clínico Universitario, Valladolid, Spain

## Case report

We describe an unusual mechanism of ischaemia and a rarely reported complication: an extrinsic coronary artery compression during percutaneous lead extraction.

**Figure 1:**
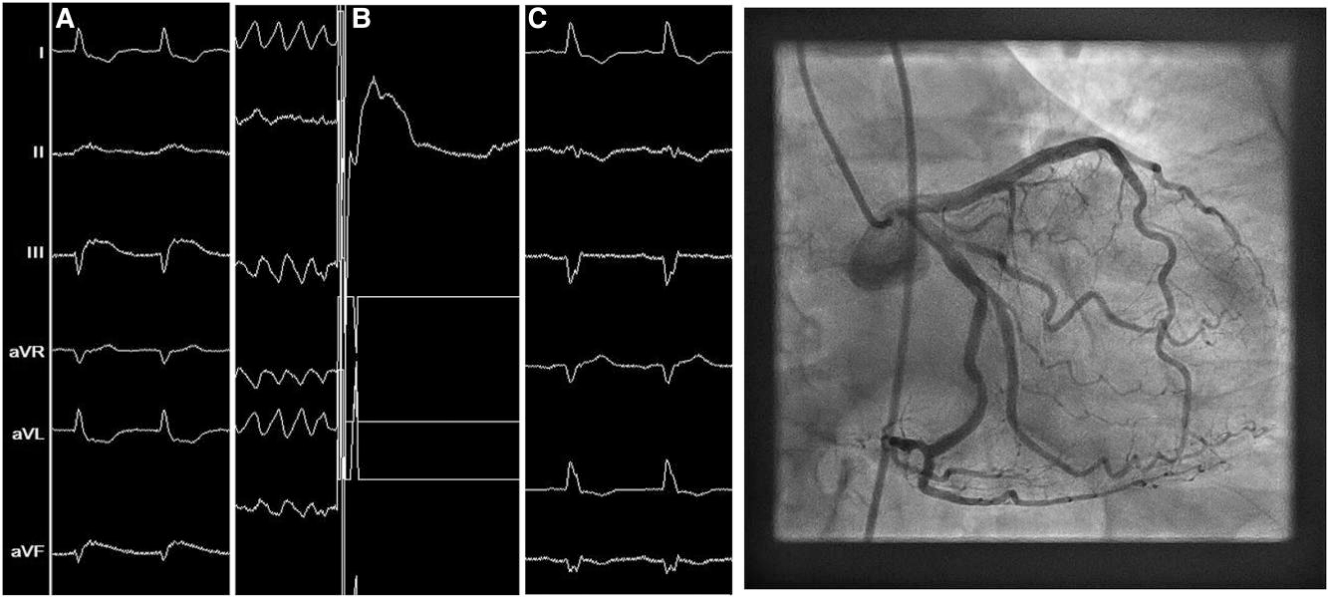
Left panel: succession of events during percutaneous coronary sinus lead extraction; (*A*) ST-segment elevation; (*B*) ventricular fibrillation and external shock; (*C*) post-extraction. Right panel: coronarography performed after the procedure.

A 67-year-old female with a history of a breast cancer diagnosed in 2011 was treated with chemotherapy and local surgery with complete resolution of the disease. In 2015, she was diagnosed with dilated cardiomyopathy with severely reduced ejection fraction secondary to chemotherapy. In 2017, despite optimal medical treatment, she remained in New York Heart Association functional Class II and had developed a left bundle branch block. Consequently, she underwent cardiac resynchronization therapy with defibrillator device implantation, with a favourable response. In 2021, she was admitted to hospital for generator exposure without signs of systemic infection. Antibiotic treatment was started. Blood and local cultures were obtained, and a transthoracic echocardiography was performed, which showed no evidence of infective endocarditis. She underwent complete extraction of the cardiac implantable device and its components.^1^ The procedure was performed percutaneously under general anaesthesia, assisted by a cardiac surgeon, guided by transoesophageal echocardiography and using an *excimer* laser system (Biomenco®). Removal of the atrial and right ventricular leads coursed without incidents. During the attempt to remove the left ventricular electrode, a transient ST-segment elevation appeared on the electrocardiogram (ECG) inferior leads, and seemed to become more pronounced each time when traction was applied. The patient’s haemodynamic status was stable during this event and the procedure was continued. Eventually, the coronary sinus lead was released, and immediately thereafter, a ventricular fibrillation was developed, which was satisfactorily terminated by an external defibrillator. Sinus rhythm was restored, and the ST-elevation completely disappeared. An urgent coronary angiography was performed shortly after the procedure which showed a left dominant system with a large circumflex artery and no coronary disease (*Figure 1* and Supplementary material online).

The patient was extubated a few hours later and no complications were noted. Post-procedure ECGs and transthoracic echocardiography did not show any changes and the patient evolved properly. She was discharged from hospital a few days later without any sequel.

We hypothesise that the mechanism of the transient ischaemia developed during the procedure was the extrinsic compression of the circumflex artery (or one of its branches) by the CS lead while pulling back, leading to dynamic changes. After complete removal of the lead and cessation of the compression, a reperfusion-induced ventricular fibrillation was developed. The condition resolved spontaneously once the lead was released and no coronary artery lesions were seen.

Percutaneous lead extraction is sometimes a high-risk procedure and many complications may occur.^2^ Probably the most feared and common are pericardial effusion, tamponade, and haemorrhage. Rare causes of acute myocardial infarction during the procedure have been described in the literature,^3^ mainly related to injury of the left anterior descending artery by the right ventricular lead, but injury of the coronary arteries by the CS lead is even more anecdotal.^4^ This case reflects a hypothetical, specific, and a very concrete mechanism of myocardial ischaemia. We hope that its description will help other groups and that it could be prevented in similar interventional procedures.

## Supplementary Material

ytad568_Supplementary_Data

## Data Availability

The data underlying this article are available in the article and in its online Supplementary material.

## Lead author biography

Gonzalo Fernandez Palacios is a graduate in Medicine from Miguel Hernandez University. He is a cardiologist at Clinic University Hospital in Valladolid.

## Supplementary material

**Consent:** The patient has given permission to publish the case following and fulfilling COPE guidelines.

**Funding:** None.
